# Heterogeneity of weight loss and transcriptomic signatures in pancreatic ductal adenocarcinoma

**DOI:** 10.1002/jcsm.13390

**Published:** 2023-12-20

**Authors:** Andrea N. Riner, Kelly M. Herremans, Vignesh Vudatha, Song Han, Xufeng Qu, Jinze Liu, Nitai Mukhopadhyay, Devon C. Freudenberger, Thomas J. George, Sarah M. Judge, Andrew R. Judge, Steven J. Hughes, Jose G. Trevino

**Affiliations:** ^1^ Department of Surgery University of Florida College of Medicine Gainesville Florida USA; ^2^ Department of Surgery Virginia Commonwealth University Richmond Virginia USA; ^3^ Department of Biostatistics Virginia Commonwealth University Richmond Virginia USA; ^4^ Department of Medicine University of Florida College of Medicine Gainesville Florida USA; ^5^ Department of Physical Therapy University of Florida Health Science Center Gainesville Florida USA

**Keywords:** Adipose tissue, Malabsorption, Nutrition, Pancreaticobiliary obstruction, Skeletal muscle, Weight loss

## Abstract

**Background:**

Pancreatic ductal adenocarcinoma (PDAC) is highly associated with cachexia and weight loss, which is driven by the tumour's effect on the body. Data are lacking on differences in these metrics based on PDAC anatomic location. We hypothesize that the primary tumour's anatomic region influences the prevalence and severity of unintentional weight loss.

**Methods:**

Treatment naïve patients with PDAC who underwent pancreatectomy at a single institution between 2012 and 2020 were identified retrospectively. Patients with pancreatic head or distal tumours were matched by sex, age, N and T stage. Serologic and anthropometric variables were obtained at the time of diagnosis. Skeletal muscle index (SMI), muscle radiation attenuation (MRA) and adiposity were measured. The primary outcome was presence of significant weight loss [>5% body weight (BW) loss in past 6 months]. Signed rank tests, Cochran Mantel Haenszel tests and Kaplan–Meier survival analysis are presented. RNA‐seq of tumours was performed to explore enriched pathways related to cachexia and weight loss.

**Results:**

Pancreatic head tumours (*n* = 24) were associated with higher prevalence (70.8% vs. 41.7%, *P* = 0.081) and degree of weight loss (7.9% vs. 2.5%, *P* = 0.014) compared to distal tumours (*n* = 24). BMI (*P* = 0.642), SMI (*P* = 0.738) and MRA (*P* = 0.478) were similar between groups. Combining BW loss, SMI and MRA into a composite score, patients with pancreatic head cancers met more criteria associated with poor prognosis (*P* = 0.142). Serum albumin (3.9 vs. 4.4 g/dL, *P* = 0.002) was lower and bilirubin (4.5 vs. 0.4 mg/dL, *P* < 0.001) were higher with pancreatic head tumours. Survival differed by tumour location (*P* = 0.014) with numerically higher median overall survival with distal tumours (11.1 vs. 21.8 months; *P* = 0.066). Transcriptomic analysis revealed inactivation of appetite stimulation, weight regulation and nutrient digestion/metabolism pathways in pancreatic head tumours.

**Conclusions:**

Resectable pancreatic head PDAC is associated with higher prevalence of significant weight loss and more poor prognosis features. Pancreaticobiliary obstruction and hypoalbuminemia in patients with head tumours suggests compounding effects of nutrient malabsorption and systemic inflammation on molecular drivers of cachexia, possibly contributing to shorter survival. Therefore, PDAC‐associated cachexia is a heterogenous syndrome, which may be influenced by the primary tumour location. Select patients with resectable pancreatic head tumours may benefit from nutritional rehabilitation to improve outcomes.

## Introduction

Cancer‐associated weight loss and cachexia (weight loss and sarcopenia) affects nearly 80% of patients with pancreatic cancer.^1^ This syndrome, at least in part driven by the cancer itself, induces a catabolic state that limits patients' ability to tolerate chemotherapy, renders them less fit for surgical resection, reduces quality of life and performance status and culminates in shorter survival.^2^, ^3^, ^4^, ^5^ Sarcopenia (low muscle mass) and myosteatosis have been associated with increased risk of complications with surgical resection of gastrointestinal tumours and overall survival.^6^, ^7^ While unintentional weight loss (UWL) is common among patients with pancreatic ductal adenocarcinoma (PDAC),^8^ this statement may oversimplify a complex disease that intersects with metabolic, inflammatory and digestive processes.

The pancreas exhibits physiologic and anatomic diversity based on regionality. Under normal physiologic conditions, regional differences in cellular density have been noted. Islet cell density increases from the pancreatic head to the tail, whereas pancreatic polypeptide (PP) cells are found preferentially in the head and uncinate.^9^ PP cells are involved in post‐prandial signalling, with levels increasing with age and diabetes.^10^ Insulin‐secreting β‐cells in the pancreatic head are preferentially depleted in patients with type 2 diabetes.^11^ This may have implications in carcinogenesis as diabetes is a known risk factor for pancreatic cancer.^12^ The pancreatic head is also subject to inflammatory changes in response to duodenal microbial reflux into the pancreatic duct.^13^ Mass effect from tumours in the head may also obstruct or constrict digestive enzyme and bile flow, impacting digestive health. PDAC may arise throughout the pancreas, with presentation and prognosis differing based on the tumour's anatomic location. Pancreatic head tumours are less insidious as they often manifest with overt signs and symptoms of pancreatobiliary obstruction, whereas distal tumours may have subtle or non‐specific symptoms in earlier stages. Distal (body/tail) pancreatic tumours are generally presumed to be more malignant and deadlier, whether due to presentation with more advanced disease and/or more aggressive tumour biology.^14^, ^15^ Molecularly, distal PDAC tumours have been associated with higher rates of KRAS and SMAD4 mutations, as well as fewer targetable alterations.^16^ Blood flow to the pancreas may be affected by tumour location in that the pancreatic blood flow index (PFI) proximal to the tumour is similar to that of a normal pancreas, whereas the PFI distal to the tumour is reduced.^17^ The propensity for lymphatic drainage to various peri‐pancreatic nodal stations is dependent upon tumour location,^18^ which may have implications on the location of distant metastases. Body and tail tumours are associated with higher rates of peritoneal metastases, as well as multiple sites of metastatic foci.^19^ Specifically, tail tumours have the highest propensity to metastasize, which has been supported by clinical and molecular studies.^14^, ^19^ Location of metastases ultimately influences survival, as isolated liver metastases are associated with significantly shorter survival compared to isolated pulmonary or distant nodal metastases.^19^, ^20^ In sum, the pancreas exhibits heterogenous anatomy and physiology by pancreatic region, and PDAC is similarly diverse in its pathophysiology based upon the tumour's anatomic location within the pancreas.

Tumours arising in different regions of the pancreas may exhibit divergent biology in addition to varying implications of mass effect, yet to our knowledge, pancreatic cancer‐induced UWL has not been investigated by stratifying patients by the anatomic region of the primary tumour. Such differences may identify opportunities to pre‐operatively manage patients accordingly. Thus, we aimed to investigate pancreatic cancer‐associated weight loss and sarcopenia by tumour anatomic location and hypothesize that anatomic region of the primary tumour influences the prevalence and severity of UWL.

## Methods

### Patient selection

**Figure 1:**
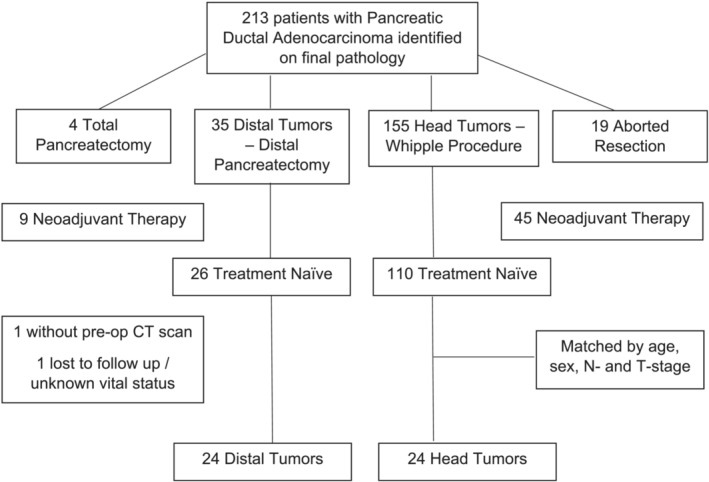
Patient selection based on eligibility criteria. Among treatment‐naïve patients who underwent distal pancreatectomy and who had pre‐operative CT imaging and known vital status, there were 24 patients with distal tumours. Treatment naïve patients who underwent a Whipple procedure for pancreatic head tumours were then matched to the 24 patients with distal tumours based on age, sex, N‐ and T‐stage.

Patients with presumed resectable PDAC who were taken to the operating room from August 2012 – December 2020 were identified retrospectively. Those who underwent pancreaticoduodenectomy or distal pancreatectomy were included in the analysis, while those who had a total pancreatectomy or were found to be unresectable intraoperatively due to advanced disease were excluded (Figure 1). Patients who underwent neoadjuvant chemotherapy were also excluded to avoid the influence of treatment on body composition and tumour transcriptomics. Based on our assumption of a large effect size (40% difference in prevalence of cachexia between groups), a minimum sample size of 23 patients in each group was determined to be adequate to achieve 80% power with alpha 0.05. There were fewer patients with distal tumours that met eligibility criteria; therefore, patients with pancreatic head tumours were 1:1 matched to patients with distal tumours. Exact matching by sex and calliper matching by age ±5 years were implemented. Nodal stage was matched exactly, with the exception of two patients who had N2 disease in the distal pancreas with no exact match from the head group available. Lastly, tumour stage was then used as a tie‐breaker to identify the closest match. This study was approved by the University of Florida Institutional Review Board (IRB 202101423).

### Patient variables

Demographic and clinical variables included age, sex, race, body mass index (BMI), per cent body weight (BW) loss in the 6 months prior to diagnosis, as well as serum albumin, bilirubin, haemoglobin and platelet count at the time of diagnosis. Computed tomography scans at the time of diagnosis were obtained and analysed using sliceOmatic ® 5.0 software (TomoVision, Magog, Canada) to compute skeletal muscle index (SMI), muscle radiation attenuation (MRA), visceral adipose tissue (VAT), subcutaneous adipose tissue (SAT) and intramuscular adipose tissue (IMAT) of axial images at the level of the L3 vertebra. Pathologic variables included tumour stage, tumour size, nodal stage, number of positive lymph nodes, lymph node positivity ratio, margin status, presence of perineural or lymphovascular invasion, and histologic differentiation. Overall survival time (months) from the date of surgery to death was calculated. Vital status was confirmed as of 15 March 2021.

### Statistical analysis

Patients were categorized into two groups, based on anatomic location of the primary tumour, classified as either head or distal pancreas. The primary outcome was presence of UWL, reported as a dichotomous variable, and defined as >5% BW loss in the 6 months prior to diagnosis.^8^, ^21^ Secondary outcomes included overall survival time and a modified composite cachexia score (0–3), which was determined by the number of poor prognostic features that were met and adapted from Martin, *et al*.^22^ These features included >5% BW loss, SMI (<41 cm^2^/m^2^ for women regardless of BMI, <43 cm^2^/m^2^ for men with BMI < 25.0 kg/m^2^ or <53 cm^2/^m^2^ for men with BMI 25.0 kg/m^2^ or greater) and MRA (<41 HU for men and women with BMI < 25.0 kg/m^2^, <33 HU for men and women with BMI 25.0 kg/m^2^ or greater) at the level of the L3 vertebra. Thresholds for SMI and MRA were founded on previously established values associated with poor prognosis.^22^ Descriptive statistics, signed rank tests (continuous), Cochran Mantel Haenszel tests (binary and ordinal) and Kaplan–Meier survival analysis are presented with statistical significance at *P* < 0.05.

### Transcriptomics

Tumour specimens were obtained from the operating room at the time of surgical resection. Tumour segments measuring approximately 5 × 5 × 5 mL were preserved in RNAlater solution and stored in liquid nitrogen. Archived tumours from 25 of 48 patients included in the clinical data analysis were retrieved and RNA was extracted by Novogene. mRNA‐Seq was performed on RNA from 15 specimens that passed quality control checks. The sequencer produced FASTQ formatted files that store RNA‐seq reads and their corresponding quality scores. We used ‘FastQC’,^23^ a quality control tool for high throughput sequencing data, to manage the data quality, and then we applied ‘BBDuk’,^24^ a reads‐trimming tool, to trim adapter sequences. Next, we used ‘STAR’^25^ (v2.7.9.a) to align the reads to the human reference genome, and then we employed ‘FeatureCounts’^26^ for counting reads to genomic features.

In order to perform differential expression analysis, we combined samples' read count and created matrices in terms of various comparisons such as ‘>5% vs. <5% BW loss’ in pancreatic head tumour samples and ‘>5% vs. <5% body weight loss’ in distal pancreatic samples. We used R (v4.0) and DESeq2 (v1.30.1) to perform differential expression analysis. The function in DESeq2 applies negative binomial GLM fitting and Wald statistics so that the statistics such as ‘log2FoldChange’ and ‘*P*‐value’ can reveal the significant up/down‐regulated differential expressed genes (DEGs) in each comparison. Genes were considered differentially expressed based on a fold change cutoff of 1.5.

For a better understanding of the biological activities among the various sample groups, we performed pathway analysis using Ingenuity Pathway Analysis (IPA)^27^ by feeding in the DEGs with fold change of 1.5 and *P*‐value of <0.05. IPA has a priori established network that exhibits how the interactions of genes are correlated with biological functions. Therefore, IPA can identify enriched pathways according to the input DEGs and make predictions about whether the pathways are activated or inhibited in patients regardless of weight loss (UWL vs. no UWL), as well as stratified by tumour location (head vs. distal). Canonical pathways, specifically those associated with cachexia, were considered significant if the absolute *Z*‐score was >2 and *P*‐value was <0.05.

## Results

**Figure 2:**
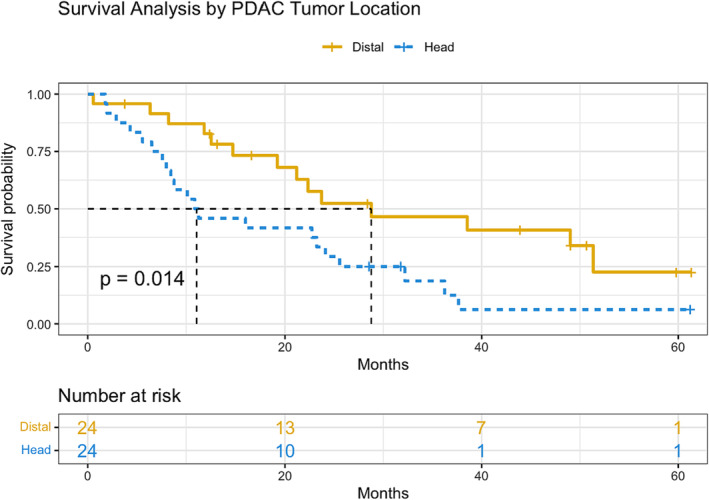
Survival analysis by PDAC tumour location. Kaplan–Meier survival analysis revealed significant differences in survival between patients with tumours in the distal pancreas compared to the head of the pancreas, with significantly longer survival among patients with PDAC in the distal pancreas (*P* = 0.014).

Twenty‐four patients with distal pancreas tumours met inclusion criteria and were matched to 24 patients with pancreatic head tumours (Figure 1). Patients were similar between the two groups based on age, sex and N‐stage due to matching (Table 1). Although more patients had higher T‐stage in the pancreatic head group, the median tumour size (cm) did not differ significantly between groups. UWL was more prevalent in patients with tumours in the head (70.8%) compared to the distal pancreas (41.7%) (*P* = 0.081) (Table 2). The median percentage of BW loss was significantly higher with head (7.9%) compared to distal tumours (2.5%) (*P* = 0.014). Despite differences in BW loss, patients in the two groups had similar BMI (26.9 vs. 28.0 kg/m^2^, *P* = 0.642), SMI (39.6 vs. 39.5 cm^2^/m^2^, *P* = 0.739), MRA (28.7 vs. 30.1 HU, *P* = 0.478) and overall adiposity (372 vs. 345 cm^2^, *P* = 0.955). When BW loss, SMI and MRA were combined into a pre‐defined modified composite score based on previously established thresholds,^22^ patients with pancreatic head tumours met more criteria associated with cachexia and poor prognosis compared to patients with distal pancreatic tumours (*P* = 0.142) (Table 2). Serum albumin (3.9 vs. 4.3 g/dL, *P* = 0.002) and total bilirubin (4.5 vs. 0.4 mg/dL, *P* < 0.001) differed between groups (Table 3). Kaplan–Meier survival analysis revealed significant differences in survival between the two groups (*P* = 0.014) and median overall survival was numerically longer with distal tumours (21.8 months) compared to head tumours (11.1 months) (*P* = 0.066) (Figure 2).

**Table 1:** Patient demographics and tumour pathology

	Head	Distal	*P*‐value
	(*N* = 24)	(*N* = 24)	
Age			
Mean (SD)	70.9 (7.01)	71.6 (6.38)	0.741
Median [Min, Max]	71.9 [55.7, 80.1]	72.2 [59.8, 84.8]	
Sex			
Female	12 (50.0%)	12 (50.0%)	1.000
Male	12 (50.0%)	12 (50.0%)	
Race			
African American	0 (0%)	3 (12.5%)	0.113
Asian	0 (0%)	1 (4.2%)	
White	24 (100%)	20 (83.3%)	
T‐Stage			
1	0 (0%)	1 (4.2%)	0.034
2	1 (4.2%)	7 (29.2%)	
3	23 (95.8%)	16 (66.7%)	
Tumour size (cm)			
Mean (SD)	3.36 (0.928)	4.19 (1.81)	0.060
Median [Min, Max]	3.40 [1.20, 5.00]	3.60 [2.10, 8.70]	
Missing	1 (4.2%)	1 (4.2%)	
N‐stage			
0	5 (20.8%)	7 (29.2%)	0.400
1	18 (75.0%)	14 (58.3%)	
2	1 (4.2%)	3 (12.5%)	
Positive lymph nodes			
Mean (SD)	4.08 (4.22)	2.04 (2.66)	0.052
Median [Min, Max]	3.00 [0, 16.0]	1.00 [0, 10.0]	
Nodal positivity ratio			
Mean (SD)	0.19 (0.21)	0.14 (0.15)	0.291
Median [Min, Max]	0.15 [0.00, 0.71]	0.08 [0.00, 0.50]	
Perineural invasion			
No	0 (0%)	3 (12.5%)	0.233
Yes	24 (100%)	21 (87.5%)	
Lymphovascular invasion			
No	6 (25.0%)	9 (37.5%)	0.533
Yes	18 (75.0%)	15 (62.5%)	
Margin status			
Negative	11 (45.8%)	21 (87.5%)	0.006
Positive	13 (54.2%)	3 (12.5%)	
Differentiation			
Well	2 (8.3%)	4 (16.7%)	0.663
Moderate	14 (58.3%)	12 (50.0%)	
Poor	8 (33.3%)	8 (33.3%)	
Undifferentiated	0 (0%)	0 (0%)	

**Table 2:** Body composition data for cachexia, sarcopenia and adiposity

	Head	Distal	*P*‐value
	(*N* = 24)	(*N* = 24)	
Cachectic (>5% BW Loss)			
No	7 (29.2%)	14 (58.3%)	0.081
Yes	17 (70.8%)	10 (41.7%)	
BW loss (%)			
Mean (SD)	10.3 (10.1)	4.4 (4.8)	0.014
Median [Min, Max]	7.9 [0, 39.2]	2.5 [0, 14.2]	
BMI			
Mean (SD)	26.6 (5.1)	27.2 (4.39)	0.642
Median [Min, Max]	26.9 [14.3, 36.1]	28.0 [18.4, 35.8]	
SMI (cm^2^/m^2^)			
Mean (SD)	41.1 (10.4)	42.0 (10.0)	0.739
Median [Min, Max]	39.6 [21.1, 62.2]	39.5 [25.7, 64.5]	
MRA (HU)			
Mean (SD)	29.9 (8.2)	31.6 (8.7)	0.478
Median [Min, Max]	28.7 [14.7, 44.0]	30.1 [17.5, 52.5]	
IMAT (cm^2^)			
Mean (SD)	9.61 (7.7)	11.3 (8.6)	0.487
Median [Min, Max]	8.1 [1.4, 39.9]	9.7 [0.2, 37.3]	
VAT (cm^2^)			
Mean (SD)	154 (68.5)	179 (120)	0.376
Median [Min, Max]	161 [3.9, 253]	162 [36.9, 407]	
SAT (cm^2^)			
Mean (SD)	215 (122)	186 (66.5)	0.314
Median [Min, Max]	213 [39.2, 493]	167 [95.0, 330]	
All adipose tissue (cm^2^)			
Mean (SD)	380 (169)	377 (156)	0.955
Median [Min, Max]	372 [44.5, 652]	345 [187, 721]	
# of criteria met			
0	0 (0%)	2 (8.3%)	0.142
1	4 (16.7%)	7 (29.2%)	
2	10 (41.7%)	11 (45.8%)	
3	10 (41.7%)	4 (16.7%)	

Measures of skeletal muscle quantity (SMI), quality (MRA) and adiposity from CT images are reported. Cachexia (>5% BW loss), SMI and MRA were combined to create a composite score reflecting the number of cachexia or sarcopenia criteria met, which serves as a surrogate for degree of cachexia.

BW, body weight; BMI, body mass index; IMAT, intramuscular adipose tissue; MRA, muscle radiation attenuation; SAT, subcutaneous adipose tissue; SMI, skeletal muscle index; VAT, visceral adipose tissue.

**Table 3:** Laboratory values related to nutritional status, biliary obstruction and bone marrow function

	Head	Distal	*P*‐value
	(*N* = 24)	(*N* = 24)	
Albumin (g/dL)			
Mean (SD)	3.7 (0.6)	4.2 (0.4)	0.002
Median [min, max]	3.9 [2.4, 4.6]	4.3 [2.9, 4.7]	
Total bilirubin (mg/dL)			
Mean (SD)	5.6 (5.0)	0.5 (0.2)	<0.001
Median [min, max]	4.5 [0.4, 15.6]	0.4 [0.2, 1.1]	
Haemoglobin (g/dL)			
Mean (SD)	12.4 (2.0)	13.3 (1.4)	0.064
Median [min, max]	12.8 [6.9, 15.4]	13.3 [10.6, 15.7]	
Platelets (x 10^9^/L)			
Mean (SD)	243 (107)	243 (88)	0.998
Median [min, max]	230 [95, 609]	230 [44, 490]	

### Transcriptomic signatures associated with cachexia

**Figure 3:**
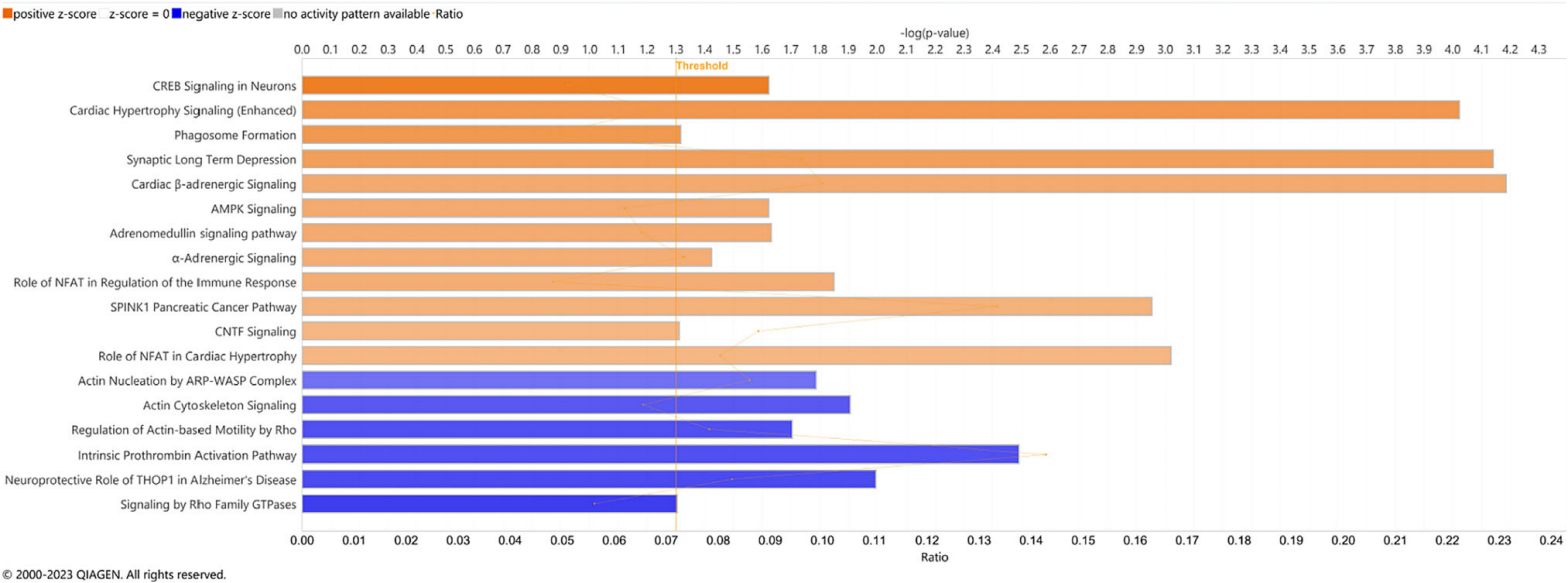
Enriched pathways in PDAC tumour transcriptomes among cachectic patients. Ingenuity Pathway Analysis (Qiagen®) data are presented for cachectic patients compared to non‐cachectic patients, regardless of tumour location. The pathways displayed represent the most inactivated (blue) and activated (orange) pathways in tumours from cachectic patients (absolute *Z*‐score of 2 or more).

Gene expression data were obtained for 15 tumours available from archived surgical specimens (UWL, head = 3; no UWL, head = 3; UWL, distal = 3; no UWL, distal = 6). Tumours from patients with UWL (*n* = 6), regardless of tumour location, were compared to those from patients without UWL (*n* = 9) to identify enriched pathways associated with cachexia. Pathways known to be involved in adipose tissue (white adipose tissue browning pathway and UVA‐Induced MAPK signalling), skeletal and cardiac muscle [androgen signalling, endothelin‐1 signalling, cardiac β‐adrenergic signalling (enhanced), and cardiac hypertrophy signalling], as well as metabolic processes (insulin secretion signalling, G‐protein coupled receptor (GPCR)‐mediated nutrient sensing in enteroendocrine cells) were significantly activated in UWL patient tumours (Figure 3, Table S1). Differentially expressed genes between tumours from UWL patients relative to non‐UWL patients (*n* = 1354) are presented in Table S2.

**Figure 4:**
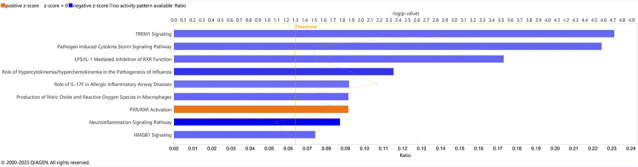
Enriched pathways in PDAC tumour transcriptomes among cachectic patients by tumour anatomic location. Ingenuity Pathway Analysis (Qiagen®) data are presented for cachectic patients with pancreatic head tumours compared to cachectic patients with distal pancreatic tumours. The pathways displayed represent the most inactivated (blue) pathways in tumours from cachectic patients (absolute Z‐score of 2 or more).

Tumours from UWL patients were then stratified by their anatomic location to assess possible differences in signalling and drivers of weight loss between head and distal PDAC tumours. Enriched pathways from head tumours relative to distal tumours were identified (Figure 4, Table S3). Activated pathways in head tumours relative to distal tumours included those with roles in tissue repair (inhibition of matrix metalloproteases), inflammatory signalling in muscle wasting (antioxidant action of vitamin C, STAT3 pathway), lipid [liver X receptors (LXR)/retinoid X receptors (RXR) activation] and muscle (neuregulin signalling) homeostasis, as well as drug and nutrient [xenobiotic metabolism constitutive active/androstane receptor (CAR) and pregnane X receptor (PXR) signalling pathways] metabolism. Inactivated pathways in head tumours relative to distal tumours included those with roles in inflammatory effects on lipid metabolism (LPS/IL‐1 mediated inhibition of RXR function), appetite stimulation and weight regulation (IL‐8 signalling), as well as hepatic fibrosis (hepatic fibrosis signalling pathway), IL‐17 and cytokine regulation (differential regulation of cytokine production in intestinal epithelial cells by IL‐17A, differential regulation of cytokine production in macrophages and T helper cells by IL‐17A and IL‐17F), wound healing and fibrosis (wound healing signalling pathway), cardiac muscle hypertrophy [cardiac hypertrophy signalling (enhanced)], and nutrient digestion/metabolism (GPCR‐mediated nutrient sensing in enteroendocrine cells, phospholipases). Differentially expressed genes between tumours from UWL patients with pancreatic head tumours relative to UWL patients with distal pancreas tumours (*n* = 1087) are presented in Table S4.

## Discussion

The evidence provided in this study supports the notion that pancreatic cancer associated weight loss is a complex process, whose manifestation is associated with the primary tumour's location. The presence of UWL regardless of primary tumour location aligns with the cachectic phenotype being driven, at least in part, by the cancer. This is further supported by our transcriptomic data revealing numerous enriched pathways implicated in cachexia for both head and distal tumours likely representing the underlying biology of tumour‐associated cachexia. When stratified by tumour location, the molecular drivers of cachexia differ, which may impact the severity of weight loss, although further investigation with a larger sample size is needed to support this. While we show a trend towards a significant difference in UWL prevalence by tumour location, the large effect size is striking, with more drastic weight loss associated with pancreatic head adenocarcinoma. Our data on hypoalbuminemia and hyperbilirubinemia suggest that the inherent drivers of cachexia in pancreatic head tumours may potentially be compounded by systemic inflammation, biliary obstruction and pancreatic exocrine insufficiency (PEI) that contribute to nutrient malabsorption, as supported by dysregulation of pathways involved in nutrient digestion/metabolism, appetite stimulation, and weight regulation, likely resulting in more profound weight loss. While absolute values in SMI and MRA were similar between groups, it is important to note that thresholds associated with shorter survival are dependent upon sex and BMI. Our modified scoring system incorporating measures of BW loss and sarcopenia accounts for these thresholds and provides a more comprehensive assessment of the severity of cachexia. Based on this composite score, patients with pancreatic head adenocarcinoma had greater severity of cachexia, with all patients meeting at least 1 criterion, whereas two patients (8%) with distal tumours that had neither significant BW loss nor sarcopenia.

Our data revealing lower serum albumin and higher bilirubin levels in patients with pancreatic head cancers suggest that these patients may experience increased levels of systemic inflammation but may also be more likely to have potentially reversible or treatable causes of nutrient malabsorption, which may have implications on outcomes. Albumin, an acute phase reactant, is among one of several factors in pre‐operative PDAC patients that is associated with early recurrence and shorter survival regardless of tumour location.^28^ Partelli *et al*. studied PEI in patients with advanced pancreatic cancer and found anaemia (haemoglobin ≤ 12 g/L), hypoalbuminemia (≤40 g/L), and ‘extremely reduced’ faecal elastase‐1 (≤20 μg/g) to be associated with significantly shorter survival.^29^ In their study, ‘extremely reduced’ faecal elastase‐1 and jaundice were more likely to be found in patients with pancreatic head tumours (*P* < 0.01 each). Although faecal elastase‐1 concentrations were not available for patients in our study, we similarly found pancreatic head tumours to be associated with significantly lower albumin, higher total bilirubin, numerically lower haemoglobin and shorter survival.

Distal tumours are thought to portend a worse prognosis due to more advanced stage at diagnosis, higher mutational burden and more harmful molecular alterations. Although distal pancreatic tumours are more likely to present at advanced stage, prior epidemiologic data demonstrated higher 3‐year overall survival for local/resectable distal tumours, consistent with our survival analysis.^15^ Regarding molecular differences, Bi *et al*. performed whole exome and RNA sequencing on surgically resected PDAC, comparing head to body/tail tumours, finding that distal tumours had higher mutational burden.^14^ Furthermore, distal cancers were associated with biological processes such as epidermal development, epidermal cell differentiation and proteolysis, whereas head cancers were associated with lipid metabolic processes. These findings suggest molecular differences in tumour biology and higher propensity for distal tumours to metastasize through epithelial to mesenchymal transition.^14^

The liver, a common site to which PDAC metastasizes, contributes to energy wasting and acute phase response in cachexia at baseline.^30^, ^31^, ^32^ The presence of liver metastases likely plays a role in development or progression of UWL and cachexia as well. In a colorectal cancer mouse model studying the effect of liver metastases, BW loss and skeletal muscle wasting were more prominent in mice with liver metastases compared to those without metastatic disease.^33^ Transcriptomic analysis of the skeletal muscle from mice with liver metastases demonstrated similarly enriched pathways as tumours from UWL patients in our study, including cardiac hypertrophy signalling, endothelin‐1 signalling and STAT3,^33^ suggesting the involvement of common upstream regulators. In PDAC, high serum IL‐6 levels have been associated with presence and volume of liver metastases, as well as reduced skeletal muscle mass.^34^ Analysis of multiple cancers within The Cancer Genome Atlas (TCGA) revealed upregulation of pathways related to cytokine interactions, complement and coagulation cascades, as well as Rap1 signalling pathways that were more specific to PDAC.^35^ Additionally, PDAC patients in TCGA had the highest average BW loss, highest prevalence of cachexia and highest number of cachexia‐inducting factors compared to other cancer types.^35^ Our transcriptomic analysis similarly identified enrichment of pathways involved in cytokine signalling, coagulation cascades, as well as other pathways previously reported in PDAC‐associated cachexia.^35^, ^36^ Despite studies of the transcriptome in PDAC‐associated cachexia and PDAC by tumour location, we are unaware of any studies that merge these two analyses to assess potential differences in molecular drivers of PDAC‐associated cachexia based on anatomic location of the primary tumour. These differences in molecular drivers may be hypothesis generating in that they could serve to identify subsets of patients in which surgical resection over nutritional optimization while undergoing neoadjuvant chemotherapy may lead to better outcomes, or vice versa, although additional studies are needed before precision medicine could be applied in this manner.

There are notable limitations of our study that warrant acknowledgement. Lead time bias may play a role in our results pertaining to skeletal muscle mass and quality, as well as adiposity. The lead time from cancer development to diagnosis is likely similar regardless of tumour location. We attempted to control for lead time by matching patients based on T‐stage and N‐stage. Beyond T‐stage, the tumour sizes, potentially a more accurate surrogate for duration of disease, were similar between the two anatomic regions. To our surprise, there were no differences in SMI, MRA and adiposity based on tumour location, although the lack of difference could be due to insufficient sample size to analyse our data stratified by sex or BMI. We attempted to adjust for this in our composite cachexia score that factored in sex and BMI into determining whether the patient met thresholds associated with poor prognosis. It is possible that patients with pancreatic head tumours causing PEI and biliary obstruction may present more acutely, which could impact weight more rapidly than measurable differences in skeletal muscle and adipose tissue. We were unable to analyse the impact of UWL on progression free survival as the heterogeneity of post‐operative management and care made it difficult to assess progression free survival retrospectively. More incomplete (R1) resections were noted in patients with pancreatic head tumours, a common outcome due to anatomic structures, which contributes to survival differences between the groups. Due to limited availability of inflammatory markers, such as CRP or ratios derived from a CBC with differential, we were unable to assess levels of systemic inflammation, a hallmark of cancer‐associated cachexia, between groups beyond serum albumin levels. Transcriptomic analysis, particularly with limited sample size, is subject to risk of false discovery. We attempted to adjust for this by implementing a more stringent *P*‐value for significance. Lastly, while the difference in UWL and the composite cachexia score trended towards significance, we cannot make any conclusive remarks regarding cachexia based on these findings. UWL on its own is not representative of the consensus definition of cachexia,^37^ which has an additional SMI requirement for patients with less severe weight loss. The composite cachexia score, while accounting for weight loss and SMI, has not been accepted as a consensus definition or validated in another study.

## Conclusion

In conclusion, cachexia and UWL are present in a significant proportion of PDAC patients regardless of tumour location; however, resectable tumours in the pancreatic head may be associated with higher prevalence of UWL, more severe weight loss and more features associated with poor prognosis. Pancreaticobiliary obstruction, PEI and lower albumin in patients with pancreatic head tumours suggests compounding effects of nutrient malabsorption and systemic inflammation on cancer‐associated cachexia, possibly contributing to shorter survival. These findings suggest that PDAC and its associated cachexia are rather heterogenous and may create opportunity to improve outcomes through nutritional prehabilitation in select patients with pancreatic head PDAC prior to surgical resection. These findings should be validated with a larger independent dataset. Furthermore, consideration should be given to controlling or adjusting for primary tumour location in future PDAC cachexia studies. Nutritional optimization may include relief of biliary obstruction and pancreatic enzyme replacement therapy (PERT), in addition to dietary modifications. However, molecular analysis of biopsy specimens may provide opportunity for a precision medicine approach to identifying a subset of tumours with drivers of cachexia for which nutritional prehabilitation may not be of benefit. Measures to address unintentional weight loss and nutritional status in patients with PDAC, particularly in the pancreatic head, could potentially improve surgical outcomes, tolerance of chemotherapy, quality of life and survival.

## Conflict of interest

All authors declare no conflicts of interest.

## Supporting information


**Table S1.** Enriched Pathways in PDAC Tumour Transcriptomes Among Cachectic Patients. Ingenuity Pathway Analysis (Qiagen®) data are presented for cachectic patients compared to non‐cachectic patients, regardless of tumour location. Pathways significantly inactivated (negative z‐score) and activated (positive z‐score) in tumours from cachectic patients are reported (p‐value ≤ 0.01 or ‐log(p‐value) ≥ 2.0).

**Table S2.** Lists of differentially expressed genes in cachectic PDAC patients compared to non‐cachectic PDAC patients (*p* < 0.05, fc < −1.5 or fc > 1.5).

**Table S3.** Enriched Pathways in PDAC Tumour Transcriptomes Among Cachectic Patients by Tumour Anatomic Location. Ingenuity Pathway Analysis (Qiagen®) data are presented for cachectic patients with pancreatic head tumours compared to cachectic patients with distal pancreatic tumours. Pathways significantly inactivated (negative z‐score) and activated (positive z‐score) in pancreatic head tumours from cachectic patients are reported (p‐value ≤ 0.01 or ‐log(p‐value) ≥ 2.0).

**Table S4.** Lists of differentially expressed genes in cachectic PDAC patients with pancreatic head tumours compared to cachectic PDAC patients with distal pancreatic tumours (*p* < 0.05, fc < −1.5 or fc > 1.5).
