# Supplementary material for: Heterogeneity of weight loss and transcriptomic signatures in pancreatic ductal adenocarcinoma
Source: J Cachexia Sarcopenia Muscle. 2023 Dec 20;15(1):149–58. doi: 10.1002/jcsm.13390 (PMC10834348; doi:10.1002/jcsm.13390)
Supplement: Supplementary file 1 — Table S1. Enriched Pathways in PDAC Tumour Transcriptomes Among Cachectic Patients. Ingenuity Pathway Analysis (Qiagen®) data are presented for cachectic patients compared to non‐cachectic patients, regardless of tumour location. Pathways significantly inactivated (negative z‐score) and activated (positive z‐score) in tumours from cachectic patients are reported (p‐value ≤ 0.01 or ‐log(p‐value) ≥ 2.0). [file JCSM-15-149-s004.docx]

| **Ingenuity Canonical Pathways** | **-log(p-value)** | **z-score** |
| --- | --- | --- |
| Neuroprotective Role of THOP1 in Alzheimer's Disease | 2.00 | -2.53 |
| Intrinsic Prothrombin Activation Pathway | 2.49 | -2.45 |
| MSP-RON Signaling In Cancer Cells Pathway | 2.89 | -1.94 |
| Ephrin Receptor Signaling | 3.55 | -1.41 |
| MSP-RON Signaling In Macrophages Pathway | 2.04 | -1.27 |
| Synaptogenesis Signaling Pathway | 2.10 | -0.78 |
| Cholecystokinin/Gastrin-mediated Signaling | 2.04 | -0.71 |
| Amyotrophic Lateral Sclerosis Signaling | 4.26 | -0.33 |
| Role of JAK family kinases in IL-6-type Cytokine Signaling | 2.76 | -0.33 |
| Phospholipase C Signaling | 2.70 | -0.33 |
| D-myo-inositol-5-phosphate Metabolism | 2.03 | -0.30 |
| Serotonin Receptor Signaling | 2.08 | 0.19 |
| Pulmonary Fibrosis Idiopathic Signaling Pathway | 2.25 | 0.22 |
| Neurovascular Coupling Signaling Pathway | 2.85 | 0.24 |
| Osteoarthritis Pathway | 2.04 | 0.30 |
| Chondroitin Sulfate Biosynthesis | 2.36 | 0.38 |
| Dermatan Sulfate Biosynthesis | 2.28 | 0.38 |
| Colorectal Cancer Metastasis Signaling | 2.13 | 0.54 |
| GP6 Signaling Pathway | 2.77 | 0.63 |
| Netrin Signaling | 3.05 | 0.71 |
| Role of JAK2 in Hormone-like Cytokine Signaling | 2.86 | 0.71 |
| Opioid Signaling Pathway | 2.00 | 0.78 |
| PI3K Signaling in B Lymphocytes | 3.29 | 0.82 |
| Dilated Cardiomyopathy Signaling Pathway | 3.09 | 0.82 |
| Dopamine-DARPP32 Feedback in cAMP Signaling | 3.52 | 0.83 |
| Role Of Osteoblasts In Rheumatoid Arthritis Signaling Pathway | 4.28 | 0.85 |
| GPCR-Mediated Nutrient Sensing in Enteroendocrine Cells | 3.05 | 0.91 |
| Systemic Lupus Erythematosus In B Cell Signaling Pathway | 2.49 | 0.91 |
|  |  |  |
| **Supplemental Table 1.** **Enriched Pathways in PDAC Tumor Transcriptomes Among Cachectic Patients.** Ingenuity Pathway Analysis (Qiagen®) data are presented for cachectic patients compared to non-cachectic patients, regardless of tumor location. Pathways significantly inactivated (negative z-score) and activated (positive z-score) in tumors from cachectic patients are reported (p-value ≤ 0.01 or -log(p-value) ≥ 2.0). | | |
