# Supplementary material for: Heterogeneity of weight loss and transcriptomic signatures in pancreatic ductal adenocarcinoma
Source: J Cachexia Sarcopenia Muscle. 2023 Dec 20;15(1):149–58. doi: 10.1002/jcsm.13390 (PMC10834348; doi:10.1002/jcsm.13390)
Supplement: Supplementary file 2 — Table S2. Lists of differentially expressed genes in cachectic PDAC patients compared to non‐cachectic PDAC patients (p < 0.05, fc < −1.5 or fc > 1.5). [file JCSM-15-149-s003.docx]

**Supplemental Table 4. Lists of differentially expressed genes in cachectic PDAC patients with pancreatic head tumors compared to cachectic PDAC patients with distal pancreatic tumors (p < 0.05, fc < -1.5 or fc > 1.5).**

| **Upregulated** | | | **Downregulated** | | |
| --- | --- | --- | --- | --- | --- |
| XIST | C8B | KCNJ6 | SLCO1B3 | MOGAT1 | MCIDAS |
| SAGE1 | MMP23A | TMEM236 | LINC01151 | ADPRHL1 | AC079804.3 |
| MIR3648-1 | LINC01474 | AGAP10P | AL133335.2 | AP002800.1 | MPZL2 |
| FABP1 | TWIST1 | H1-4 | AC018695.6 | C8orf89 | PRRG4 |
| CSAG3 | ZFP57 | AL118508.1 | MUC21 | WFDC2 | SIX4 |
| HOXC11 | CCL13 | NANOS1 | SMILR | MAB21L3 | RAB3D |
| HOXC10 | AL121761.1 | AL031595.3 | GIP | AQP3 | AC008555.4 |
| RHAG | MIR4500HG | ETV7 | GALR1 | EDAR | AC036214.2 |
| CCL7 | AL731733.1 | COL8A2 | PSPHP1 | SYTL5 | CRACR2A |
| GABRA3 | CDX1 | SCG5 | TXNDC8 | STK32A-AS1 | SOX10 |
| AC006206.2 | SLC5A2 | RGS11 | AC093809.1 | DPP10-AS1 | PILRB |
| CDH16 | OPRM1 | RFX6 | SH3TC2-DT | AC137936.2 | FAT2 |
| OR5AC4P | TTR | TOGARAM2 | SPRR2A | IGLV3-12 | FREM2 |
| AC113346.1 | IGLV2-18 | COL10A1 | MAGEC3 | COL4A3 | IGHV3-53 |
| TSIX | AP000229.1 | TNFRSF11B | CLCA3P | ANGPTL7 | ANK1 |
| LINC01606 | TRPM2-AS | AL031600.1 | SLCO1B1 | MTHFD2P7 | STEAP3 |
| BARX1 | CHRNA1 | LCNL1 | CYP4Z1 | HSPD1P6 | FSIP2 |
| CALML5 | GSDMA | COMP | MIR205HG | TCL1A | TRIB1 |
| LGALS7 | AL023806.3 | AL031118.1 | LINC02742 | PRSS51 | NRG4 |
| PNMA6B | HYMAI | CDKL2 | SERPINB4 | CNTNAP3 | TP73 |
| ALPI | TKTL1 | AL139246.2 | AC005833.1 | CADM3 | AP1S3 |
| AC084121.2 | AC007496.3 | SH3D21 | CYP24A1 | CDH19 | SERPINB9P1 |
| AC025252.2 | CRYBA2 | PLPP4 | AP002478.1 | ZNF536 | RPSAP54 |
| AC036111.1 | SPATA21 | COX7CP1 | LINC02551 | TMPRSS11E | KCNJ14 |
| OBP2A | AC079416.2 | AC093895.1 | FAM201B | ZBTB16 | SLC38A1 |
| AC093367.1 | FSTL5 | VAX2 | TMEM40 | UPK3B | F3 |
| AC079753.1 | IHO1 | AC026801.2 | MIR2117HG | CYP4F35P | MYOM3 |
| AC069549.2 | LINC01506 | COL11A1 | AC146944.1 | ENDOU | CARD11 |
| AC092445.1 | NKX2-5 | FAM43B | CYP3A7 | KLK11 | PANX2 |
| RPS18P13 | GPAA1P2 | RNY4P10 | SLC34A1 | AC098850.1 | RIBC2 |
| AC006262.2 | CYP2T3P | IGSF6 | AC008687.2 | CEACAM6 | SLC7A11 |
| C1orf158 | AC004623.1 | AL355987.4 | IGHV5-10-1 | KRT15 | SYBU |
| HTR1A | AC008763.1 | SIX3 | LINC01812 | KRT7 | CATSPERB |
| GNGT1 | SLC5A12 | CLEC18A | TBC1D3D | PTX3 | PRG4 |
| HNRNPA1P68 | AC127070.1 | RBFOX3 | AC136443.1 | PPP4R4 | UHRF1 |
| MFAP1P1 | PSME2P1 | AL121933.2 | AL135784.1 | CCDC144A | MPV17L |
| PYDC1 | CACNG8 | IGKV3D-7 | DLX2 | SCUBE1 | THRB |
| IGFL2-AS1 | CFC1 | HOXC9 | LINC01793 | AC103718.1 | AC104964.3 |
| RABGAP1L-IT1 | AC010533.1 | TMEM52B | AC092675.1 | CLDN10-AS1 | FLG |
| AC026725.1 | KCNK16 | TNF | AP001885.2 | AC100858.2 | BIK |
| CR392039.1 | COX6B2 | RPLP0P2 | PGC | PLP1 | FAM91A3P |
| RN7SL23P | TMEM158 | SPATA17 | Z94277.1 | FIBCD1 | COL4A6 |
| AL133259.1 | LRRC15 | AC116407.2 | NUTM2E | PAX5 | SPTBN2 |
| AL359881.1 | AC105429.1 | GUSBP2 | AL031587.1 | MYOCD | CNTNAP3P2 |
| AL121601.2 | SOX11 | CXCL14 | AC139491.1 | LYVE1 | SLC66A1L |
| VN2R19P | AJAP1 | BX284668.5 | AC104024.3 | S100A14 | FLACC1 |
| NCBP2L | LINC02575 | RSAD2 | MUCL3 | DNER | JAG2 |
| AC078860.2 | MAFA | SPACA6 | AC020779.2 | TNNI2 | SERPINE1 |
| AC060814.2 | SSTR5 | OXT | AC079061.1 | PDK4 | TKT |
| AC120349.1 | CCL4L2 | WNT4 | LINC00940 | IRX5 | FNBP1P1 |
| TCF24 | PLAC4 | BCL2A1 | AGGF1P1 | UPK1B | CDC25A |
| RPL7P7 | AC099521.2 | C5orf46 | IGHV3-64D | CACNG4 | BCAN |
| SLC6A15 | FGF20 | GATA6-AS1 | RFPL4B | AHNAK2 | KYNU |
| LINC00114 | PAEP | PRELP | LINC02055 | AGGF1P2 | PLA2G4A |
| AL627232.1 | AL139246.4 | ATP6V0D2 | AC005786.4 | CADM2 | LINC01232 |
| Y_RNA | AL353583.1 | CBR3-AS1 | NTF4 | GPR15 | TGM2 |
| AC068134.2 | RNU6-611P | OSCAR | CYP4Z2P | MALL | PRR19 |
| ATP5MC2P4 | AL732372.2 | IL23A | AC013400.1 | SYT8 | FENDRR |
| GRAMD4P8 | DNAJC19P5 | LINC02649 | HSPD1P9 | LDB3 | C6orf223 |
| AC007485.1 | TMEM130 | SUGCT | AL732437.2 | ADH6 | KCNMB4 |
| SNORD13D | FGD5P1 | CORIN | AP001053.1 | CYP4B1 | LINC01123 |
| AC097493.1 | CHIT1 | PNMA6A | MSLNL | FIRRE | RAB38 |
| DUSP8P1 | AC068473.4 | PLCH2 | CGB5 | TMEM252 | DDIT4 |
| AC024619.3 | AC100788.2 | ABHD1 | PLA2G2A | TPSD1 | FLG-AS1 |
| FP671120.2 | IRX2 | YEATS2-AS1 | AC016405.2 | CA12 | PFKP |
| IGFL1P1 | LINC01563 | PER3 | LINC00964 | KLK7 | STON2 |
| AC005014.1 | AL137244.1 | SLC47A1 | PRB3 | AC098850.3 | ZXDB |
| AC011443.1 | AC080188.2 | EGFL6 | CD200R1L-AS1 | PPP2R2C | PRR34-AS1 |
| DYNLT3P2 | IGFBP7-AS1 | EFCC1 | ABCB11 | AC110792.3 | TUBA4A |
| MIR320B2 | AC007608.1 | ARVCF | AL591441.1 | MADCAM1 | LRP8 |
| AC008147.3 | SLITRK5 | CELF5 | AP000787.2 | FAM246C | CPD |
| AC131944.1 | TTLL10-AS1 | MTCO2P12 | AC005899.7 | JPH1 | IL12RB2 |
| LRRC3B | AL009176.1 | TSPYL5 | AP003472.1 | CTB-178M22.2 | OSBP2 |
| LINC01208 | BX322234.1 | VSNL1 | CSMD3 | ERVMER34-1 | FBXL19-AS1 |
| KRT79 | LINC01141 | NOX4 | ABCA12 | SOX7 | RAPGEF3 |
| Z98259.1 | AL136379.1 | RRAD | UGT1A8 | ARL4D | C2orf88 |
| AF230666.2 | UCN3 | GDPD3 | AC079922.1 | PCP4L1 | AFAP1L2 |
| FER1L6-AS2 | AC008429.1 | CD300LF | BNIP3P4 | EMILIN3 | IL1RL1 |
| UNC93B7 | AC023282.1 | RAB3IL1 | LINC01926 | AKR1C1 | FBXL18 |
| LINC02050 | LINC01686 | GLTPD2 | AL355482.1 | CHST2 | GAREM1 |
| AC092919.1 | SLC22A16 | AC111197.1 | DEFB4A | TRHDE | COL4A5 |
| AC008897.3 | KEL | CSPG4P10 | C5orf52 | CLEC18B | ITGA6 |
| C18orf15 | FEV | TMDD1 | GSDMC | SLC7A5 | THSD7A |
| AC073283.2 | SLC22A11 | AC005856.1 | MB | ZPLD1 | P2RX5 |
| AL591518.1 | CEP83-DT | AUXG01000058.1 | RPE65 | AC023794.4 | DNAJC12 |
| AL512430.1 | ST8SIA2 | HSD17B7P2 | VSIG10L2 | AC011503.2 | RHOBTB2 |
| BTNL3 | AL390957.1 | CNTN1 | C5orf66-AS1 | GINS4 | SLC35G2 |
| AP001062.2 | SNORA73B | RGS4 | CASC8 | KLHL30 | TWNK |
| AC103740.2 | HS3ST3A1 | MYH7B | AC007278.2 | LUZP2 | RASA4 |
| PLA2G2F | AC090877.2 | TNFRSF4 | NKX6-2 | ADAMTS15 | AVPI1 |
| AC104457.1 | CFAP65 | AC245052.4 | AC245041.1 | AL390198.1 | SOWAHC |
| AC244034.3 | C1QTNF12 | IL1B | LINC02814 | ANKRD20A5P | ZNF367 |
| SNORD3A | SMTNL1 | AEBP1 | AC008687.3 | AL358334.2 | VLDLR |
| RPS2P16 | AC114296.1 | PLD6 | AC083973.1 | TRPM6 | PLTP |
| AC090001.1 | STYXL2 | DBP | KCNA7 | PKD1P2 | MAST4 |
| ISM1-AS1 | IGHG4 | HOPX | TCN1 | INSC | SDK2 |
| C19orf84 | AC004233.4 | HOXC6 | CGB8 | ALDH1A2 | RHOD |
| AC009542.1 | IFIT1 | APCDD1 | CYP2B6 | ACSM4 | ELOVL7 |
| LINC00858 | CXCL8 | MAPK8IP2 | LINC02635 | AC005077.4 | DPY19L1 |
| GOLGA8IP | LRFN2 | TNFRSF9 | DKK1 | RAET1G | TRAM1 |
| AC093772.1 | PRSS30P | KLHDC7B | AKR1C2 | ITGB6 | NAMPT |
| AL590705.1 | MARCO | PDE8B | EVX1 | AC044802.1 | PARP4 |
| AC007014.2 | AC007292.1 | RND2 | SLC9A4 | LANCL3 | SEMA3F |
| AL133371.3 | LINC01978 | ATP6V1C2 | KLK6 | ERMP1 | PRELID1P5 |
| APOA1 | ACTN1-AS1 | AC110285.2 | IL31RA | CTSG | INPP4B |
| CHST5 | ADRA2C | FOXF2 | AC092153.1 | FGA | ACSL5 |
| CCDC13 | ANKRD1 | REEP2 | MSLN | EFNA5 | IRS2 |
| AC011465.1 | AC012531.1 | ELFN1 | GRM5 | MTAPP2 | LINC01569 |
| SLC25A48 | AC112721.2 | CLN3 | PCDHGB1 | GATA2-AS1 | HAUS6 |
| DNASE2B | PTP4A2P2 | PKDREJ | MMP1 | HP | NAMPTP1 |
| AC009133.2 | PROK2 | CNIH3 | AL138826.1 | LINCR-0001 | AL157935.1 |
| AC087741.2 | AC063960.2 | ATAD3B | PAICSP4 | HGD | EPAS1 |
| MIR325HG | NETO1 | MICA | EN2 | NMRAL2P | BEGAIN |
| MMP8 | LINC02600 | TMEM200A | C16orf74 | ATAD2 | MARS2 |
| B3GALNT1P1 | TNFSF18 | PRICKLE1 | C17orf99 | SLPI | ALPL |
| DLL3 | TTBK1 | IL4I1 | SPX | GFRA1 | L1CAM |
| TMEFF1 | MYO18B | PGR | AC013268.4 | S1PR5 | CCZ1 |
| KRT8P49 | C2orf91 | HSD17B14 | AC090192.2 | DTHD1 | REPS2 |
| HMGB2P1 | CEP295NL | ADAMTSL2 | PLA2G12AP1 | STK32A | AC093627.4 |
| BX322635.1 | AGAP7P | CDK11A | ANGPTL4 | ASPHD1 | CLN8 |
| AL662797.1 | SCO2 | GUCA1B | CYP4F60P | GAPDHP1 | GALNT12 |
| CYP26A1 | UBE2QL1 | TBX3 | PHACTR3 | MGST1 | FAIM |
| TMEM82 | GDNF-AS1 | HK3 | CRABP1 | TFCP2L1 | RNF145 |
| UBE2CP3 | RGS16 | MSI1 | RNASE7 | CHRM3 | TRIM66 |
| EMX1 | TNFRSF14-AS1 | LINC01094 | AC092691.1 | SLC22A3 | DUSP7 |
| AC010636.1 | AC027288.3 | EFCAB2 | LPAR3 | MTSS1 | GSTP1 |
| FAM209B | DIRC3 | SYNDIG1 | CP | FOXJ1 | AL078621.1 |
| TRAV2 | NBAT1 | SHF | WNT7B | CNTNAP3B | SERINC5 |
| TBX5-AS1 | LINC01762 | GLRB | LY6D | ARHGAP23 | AC010186.3 |
| AC105411.1 | CX3CR1 | ICAM1 | DES | SHISA3 | GFOD1 |
| PHGR1 | HSPA1B | ANGPTL2 | CLCA2 | LINC01348 | ZC3H12C |
| AC007663.3 | PCSK2 | SPHK1 | MOBP | ABLIM3 | PPP1R14BP3 |
| Metazoa_SRP | MMP11 | TRPV2 | CDK7P1 | ARNILA | SYTL1 |
| AL354833.2 | LY6H | SLC22A17 | B3GNT6 | AMIGO2 | LYPD5 |
| AC013643.3 | YBX2 | TRPM2 | LINC02870 | PPP1R14C | NIBAN1 |
| ALOX12B | WNT2 | MXRA8 | AC084212.1 | DNAJB13 | PLCB1 |
| LINC02712 | NKD2 | CHASERR | AC005586.1 | AL512274.1 | TMEM154 |
| GNG8 | MMP12 | ANTXR1 | CGB7 | CELSR1 | BMERB1 |
| HOXC-AS2 | PRND | MEGF6 | AC133528.1 | LRRN1 | PRKCA |
| AC018865.2 | SPIB | ITGAM | SLC5A7 | PCDHB8 | GALNT2 |
| SLC6A19 | ISL1 | QPCT | CPP | TNC | LYAR |
| TPRXL | SPATA31C2 | ITGAX | AL391152.1 | ID1 | MID1 |
| AC005086.2 | STRA6 | NFKBIE | ARSF | TRHDE-AS1 | FAM241A |
| AC004817.5 | NOTUM | CISH | RPS20P14 | NRG1 | SLC35C1 |
| AL162430.1 | SH3GL1P2 | PELATON | ANKRD20A7P | ZDHHC11 | DTNB |
| PRKCG | AL138479.2 | DKK3 | AC069218.1 | SOX2 | NOMO2 |
| CSMD1 | SNX18P12 | NCF2 | THEGL | KIF20A | CROT |
| AP007216.2 | ST8SIA3 | UAP1L1 | MYPN | AL118505.1 | DOCK9 |
| RN7SL81P | RN7SL1 | LINC00680 | FGL1 | FKBP5 | HIPK2 |
| AC145285.1 | GCKR | GAL3ST4 | CNTNAP5 | CHST6 | PLEKHA8P1 |
| VSTM2A | NKX3-2 | AC010618.3 | AC245041.2 | FOSL1 | SPTSSA |
| NLRP7 | SLC35D3 | CCNL2 | SCNN1B | PPL | PGM1 |
| SSTR5-AS1 | IGLV1-36 | ROBO3 | FAM95B1 | PSAT1 | TMEM245 |
| AP003392.5 | RN7SL3 | PGF | CNTNAP3C | AC019069.1 | IL18R1 |
| MIR5010 | HLA-DRB5 | CFAP410 | AC243830.2 | MAOA | SLC23A2 |
| AL445490.1 | ISG15 | LAYN | PMP2 | HAS2 | ITPKC |
| FRMD1 | AC010478.1 | TMEM204 | P2RY2 | AC005831.1 | FGD4 |
| Y_RNA | TSPEAR-AS1 | ISLR | FCRL1 | CCNYL5 | FGFRL1 |
| AL591501.1 | AC006460.1 | FNDC10 | HTR3A | MMP10 | FAM53B |
| ITPRIP-AS1 | FTCD | HAND2-AS1 | FGFBP1 | METTL7B | NOMO1 |
| KIF26B-AS1 | AC011352.3 | UBXN11 | IGHV2-70 | EGFR | SLC44A3-AS1 |
| ZNF709 | IL17RB | AL133216.2 | MKRN9P | AC112777.1 | RMI1 |
| CCDC92B | AL078587.2 | AC092279.1 | XPNPEP2 | GPT2 | LINC00963 |
| C9orf135 | ZNF165 | ZNF571 | RXFP1 | VSTM2L | CHMP1B |
| GJA3 | ASLP1 | NBL1 | AC002401.4 | LINC01503 | AC078819.1 |
| AP001625.2 | PTPRD-AS1 | PRANCR | AC117498.2 | PGM2L1 | PSTPIP2 |
| FP236383.5 | WASHC1 | GLIS2 | ACTN3 | PPP1R16B | UBN1 |
| CCL3L3 | BCO1 | ATG16L2 | AL354707.1 | SYT9 | COBLL1 |
| LINC01929 | AL356417.3 | C21orf58 | CHRDL2 | MYC | CYB561D1 |
| AC120036.3 | PLPPR5 | KLF3-AS1 | LINC02688 | HYAL1 | TMEM185B |
| REN | AL121723.1 | AC097376.3 | CNTNAP2 | SLC7A1 | SSH2 |
| IGFL2 | AGMO | KDM6A | CSF3 | C22orf23 | PDPK1 |
| DPY19L2P4 | ZNF295-AS1 | PNPLA7 | GPR20 | AC027117.1 | MGAT5 |
| AP000345.2 | CCDC89 | DOP1B |  |  |  |
| AC068279.2 | EGR2 | STAT2 |  |  |  |
| AC090912.1 | PTPRT | ZNF577 |  |  |  |
| AC002558.1 | AC090181.3 | U2AF1L4 |  |  |  |
| MADD-AS1 | SGIP1 | DFFB |  |  |  |
| AC241377.3 | MEOX1 | FOXP4 |  |  |  |
| COL9A3 | OPRD1 | DTX3 |  |  |  |
| APELA | AL355075.2 | MIIP |  |  |  |
| NLRP2 | CCL2 | MIB2 |  |  |  |
| RPS2P36 | AL139349.1 | MINAR1 |  |  |  |
| MYRFL | CALB2 | NCEH1 |  |  |  |
| IAPP | SYT5 | FLCN |  |  |  |
| AC009927.1 | PCSK1N | AC026412.1 |  |  |  |
| SCARNA21 | OLR1 | WDR19 |  |  |  |
| GPR119 | CFAP46 | MAN2C1 |  |  |  |
| TCERG1L | DCSTAMP | GALK2 |  |  |  |
| CCL3 |  |  |  |  |  |
