# Supplementary material for: Heterogeneity of weight loss and transcriptomic signatures in pancreatic ductal adenocarcinoma
Source: J Cachexia Sarcopenia Muscle. 2023 Dec 20;15(1):149–58. doi: 10.1002/jcsm.13390 (PMC10834348; doi:10.1002/jcsm.13390)
Supplement: Supplementary file 3 — Table S3. Enriched Pathways in PDAC Tumour Transcriptomes Among Cachectic Patients by Tumour Anatomic Location. Ingenuity Pathway Analysis (Qiagen®) data are presented for cachectic patients with pancreatic head tumours compared to cachectic patients with distal pancreatic tumours. Pathways significantly inactivated (negative z‐score) and activated (positive z‐score) in pancreatic head tumours from cachectic patients are reported (p‐value ≤ 0.01 or ‐log(p‐value) ≥ 2.0). [file JCSM-15-149-s001.docx]

**Supplemental Table 2. Lists of differentially expressed genes in cachectic PDAC patients compared to non-cachectic PDAC patients (p < 0.05, fc < -1.5 or fc > 1.5).**

| **Upregulated** | | | **Downregulated** | | |
| --- | --- | --- | --- | --- | --- |
| RPTN | HLA-K | WFIKKN1 | ADIPOQ | UNC5D | AJ009632.2 |
| CRCT1 | LINC02244 | H2BC8 | FABP1 | AC009244.1 | KANSL1L-AS1 |
| SEC61G-DT | AL590714.1 | GATA2-AS1 | CIDEA | AC090607.1 | KRT7-AS |
| IGHV1-69-2 | SULT2B1 | ARHGEF4 | B4GALNT2 | GPR79 | MROCKI |
| KLK5 | MSLN | SPINT1 | TRARG1 | DUOX2 | NUGGC |
| KRTAP4-1 | KRT17 | MT2A | GKN2 | NCAM1-AS1 | B3GAT1 |
| LINC02253 | Z97192.1 | SLC35E4 | SLC7A10 | CCL19 | AFF3 |
| CGB3 | AL844908.1 | AC025161.1 | Z97056.2 | KLRK1 | AC125494.1 |
| GGT8P | S100A11P2 | MAMDC4 | FABP2 | SUMO2P19 | CEP57L1P1 |
| CWH43 | CYP4F12 | IL11 | THRSP | AP000688.1 | TMTC1 |
| CCDC190 | ZNF474 | RNASET2 | PLIN1 | EBAG9P1 | PCDH18 |
| AL135937.1 | AQP6 | EMP3 | LBP | SLC2A3P2 | BX322639.1 |
| LINC01887 | ANPEP | ANXA3 | CLCA1 | RNF182 | AC012020.1 |
| UPK3B | MARCO | C19orf57 | KRT20 | BEND4 | RORB |
| AC011468.3 | RIMBP3B | PRRX2 | FAR2P4 | GPR182 | KLRD1 |
| AC087857.1 | Z98257.1 | KRT8P45 | TBX20 | AC104758.2 | PGAP1 |
| MICOS10P3 | SIX2 | KRT8 | IGKV6D-21 | ENPP6 | AL050343.2 |
| DSC1 | KRT14 | FAM160A1-DT | LINC00298 | FAM238A | SLAMF1 |
| AL445487.1 | AL355312.3 | MTCO1P12 | IGKV2D-28 | AC131568.1 | LINC02447 |
| HS3ST6 | RPL12P14 | PCDHGB6 | LINC02672 | TKTL1 | ALG1L15P |
| LINC01940 | EDAR | TMIGD3 | IGHV3-41 | CHRDL1 | SHC4 |
| SLURP2 | MIR200A | MYOM3 | GHRHR | NIBAN3 | SCHIP1 |
| RAB42P1 | H4C5 | TEDC1 | PTGER4P2 | AC112694.2 | KCNA5 |
| AC007688.3 | GJB2 | NAPRT | AL358075.1 | MALRD1 | SNURF |
| AC004808.2 | MYHAS | DPF1 | AL160191.3 | AC009133.3 | NRK |
| MIR4783 | IRX3 | TOB1 | AC110769.1 | SLC38A11 | GNAT2 |
| LINC01254 | MT1P3 | PAK6 | AL049695.1 | AL713852.1 | LIMCH1 |
| AL731533.3 | ULBP2 | CCND1 | AC002546.1 | LINC01781 | CASP12 |
| SLCO1B3-SLCO1B7 | AC009236.1 | PCSK6 | RNU6-91P | DLG2 | AL031432.4 |
| AL034397.1 | TCF24 | AC005785.1 | AC130448.2 | AC097369.4 | HELLPAR |
| PCED1CP | LGALS9B | BAIAP2 | NANOGP4 | FRG1KP | SRGAP3 |
| KLK6 | XXYLT1-AS2 | AC018816.1 | AL355482.1 | IGHJ5 | SVEP1 |
| AL121832.1 | ASPG | LOXL2 | AC005414.1 | ABCD2 | CMYA5 |
| AC112487.1 | RPL23AP49 | NDUFA4L2 | AL050350.1 | RBM11 | AC079921.2 |
| NPSR1 | UCA1 | UFSP1 | Y_RNA | PDGFRL2P | PRTG |
| AC108451.2 | UNC5A | C15orf62 | AC015909.3 | TNIP3 | PAPLN |
| SCEL | ANKFN1 | CD82 | AC019270.1 | AL928711.1 | PDE7B |
| AL360081.1 | AC004895.1 | MANEAL | PSG4 | DNM1P47 | AC008467.1 |
| AC090164.4 | AL354919.2 | AC004057.1 | NFILZ | NSA2P5 | LPL |
| KRT16P6 | MMP12 | GDPD3 | ARID3C | AC104964.3 | SLC1A7 |
| AC007683.3 | NGEF | AC022613.2 | FRMD1 | VEGFD | P2RX5 |
| MIR149 | MIR200B | EPHA2 | IGKV2-30 | SLC19A3 | DNM3 |
| AL592295.2 | POPDC3 | SYTL1 | ERLNC1 | ADRA1A | ITPR1 |
| AC093702.1 | AC069200.1 | AC009133.1 | AC099684.2 | SHISA3 | TG |
| TMEM244 | LINC00525 | RPS6KL1 | LINC02197 | GPR15 | PEG13 |
| MYO16-AS1 | S100A8 | VAX2 | SMIM2 | GPD1 | AC005070.3 |
| LRRC3C | PPP2R2C | HSD17B1 | PRKAR2B-AS1 | TRAV21 | EBF3 |
| AC025062.2 | AC015660.1 | H2AC20 | AC074091.1 | PTGDR | LINC01801 |
| AL136987.1 | CTAGE6 | OAZ3 | CRISP3 | CDC27P1 | ABCA10 |
| AL590644.2 | AC114810.1 | CASP9 | AL121759.1 | MYO18B | MICU3 |
| PRSS29P | SLC6A11 | S100A6 | AC025822.2 | PDZRN4 | DCLK1 |
| AC084864.2 | AC126544.2 | PSORS1C1 | RN7SKP198 | AL353795.3 | ZNF572 |
| TMSB10P1 | AC019117.2 | POTEJ | AC093010.1 | CACNB4 | OTOAP1 |
| EGLN3-AS1 | CALB2 | AC067930.5 | IGHV3-19 | VNN2 | IPCEF1 |
| LINC01873 | AP000344.2 | EXTL1 | THEGL | TRIM55 | ZBED3-AS1 |
| KLK10 | IRX5 | AC010894.1 | AL359636.2 | NLGN1 | ZNF826P |
| NDUFB1P1 | FLJ12825 | GPRC5D-AS1 | FAM9C | IGHJ3P | BMPR1AP1 |
| FGFBP1 | CFAP45 | BMP4 | AC007998.4 | PCDHB8 | GUCY1A2 |
| AC026624.1 | PLAAT2 | S100A16 | AC096647.1 | RIMS2 | AC036214.2 |
| EFCAB6-AS1 | LINC02827 | ARHGAP27P1-BPTFP1-KPNA2P3 | POU4F1 | AL353763.1 | ADAMTS1 |
| KRT78 | AL136164.3 | TRIM16L | MICD | CACNA1G | CDON |
| SEPTIN7P8 | PKMP1 | HSF4 | IGHV3-22 | PLCD4 | KCNJ2-AS1 |
| KLK7 | TBX6 | GPC1 | IGHV3-13 | LINC01934 | GYPE |
| FOXE1 | S100A9 | TCIRG1 | SLC5A7 | ANKRD36BP2 | MEGF11 |
| CGB5 | AC009097.2 | CACNB1 | TRAV10 | AC009093.3 | AC245060.6 |
| LINC02178 | CFAP73 | PPP1R14B | RTP5 | ACSL6 | AC016705.2 |
| KLK8 | SLCO4A1 | RAB40C | LTF | IGKV3-7 | ZBTB20 |
| LY6G6C | GJC2 | STYXL1 | GRID1-AS1 | AL121821.2 | IL7R |
| AC011483.1 | SIK1B | EPS8L1 | AC062004.1 | HS3ST5 | CLNK |
| AC110813.1 | LAMC2 | EBPL | IGLC7 | ZNF831 | RGS22 |
| LINC02188 | ATP2A1-AS1 | PRSS16 | RN7SKP26 | CCNP | AC083798.2 |
| AGBL1 | MT1E | TNFRSF12A | TEX35 | AC090425.2 | LRRK2-DT |
| ESRRAP1 | SMIM5 | GOLGA2P10 | IGHV3-69-1 | CU634019.1 | IGDCC4 |
| MIR657 | FP236383.5 | FBXW4P1 | IGLV3-12 | AC009630.1 | LIPE |
| AC073578.1 | AP000679.1 | RPP40 | IGHV3-64 | RGS7BP | FAT4 |
| DDX18P4 | AC010319.3 | AC132812.1 | AC243654.3 | RASL11B | RAB30 |
| AC083841.1 | GRIN3B | SDC4 | TNMD | DHDH | JPH4 |
| SLCO1B7 | ALG1L | CTSD | AC117453.1 | AC016582.1 | AC136475.1 |
| ALPP | LINC02610 | MTND6P4 | PARM1-AS1 | AL021068.1 | OSR2 |
| LINC01283 | GUCY1B2 | C1orf53 | LINC01099 | IL17REL | FAM162B |
| Y_RNA | SNORA31 | PORCN | AC133041.1 | AC010624.3 | TXLNB |
| MIR4300HG | BCO1 | WARS1 | CNTFR | AL035411.3 | GRAP2 |
| AL157931.1 | CDKN2A | BCAT1 | WEE2 | CTSG | ABCC9 |
| WNT10A | TENT5B | AK5 | PDSS1P1 | CACNA1E | AP001330.4 |
| MAP3K19 | CYP4F3 | ABCA7 | AC004948.1 | CA8 | PRUNE2 |
| SERBP1P3 | AC129507.4 | LINC01943 | AC246787.2 | SLC30A10 | GHR |
| A2ML1 | ECM1 | SQOR | YAP1P1 | LINC00582 | GCNT1 |
| MIR6814 | CETN4P | S100A11 | CD200R1L-AS1 | CCNYL6 | SMAD9 |
| RAET1L | PCP2 | AC092718.4 | IGHA2 | COL4A3 | SH3GL1P1 |
| AC004990.1 | ZNF185 | CLCF1 | SNX29P2 | ACAP2-IT1 | AKAP6 |
| IGFL1 | CYSRT1 | S100A10 | AC093158.1 | AL121985.1 | SLC2A4 |
| LINC02783 | LGR6 | P4HA2 | IGKV2D-30 | AC002451.1 | AR |
| CCK | TMEM191B | MT-ND6 | MIR9-1HG | ATP1A2 | PLCB4 |
| POU6F2-AS2 | LINC00460 | LGALS1 | IGKV1-12 | Y_RNA | MIR99AHG |
| LINC00160 | SMPD3 | CGN | TRAV1-1 | SLIT3 | RNF180 |
| MYL2 | CDRT1 | PCLAF | NWD2 | SLFN14 | ZNF66 |
| RP1 | CHAC1 | ZCWPW1 | PRRT4 | AC135352.1 | MMP16 |
| ZBED2 | LINC01843 | CAP1P2 | AL021937.3 | AC106820.4 | AL158055.1 |
| TEKT1 | VSTM2L | P4HB | TMEM82 | TSPEAR-AS2 | IL18R1 |
| MKRN4P | GPA33 | LINC01301 | CARTPT | COL14A1 | CDKN1C |
| H2BC9 | AL355472.3 | AC009237.14 | UBE2U | AL136084.2 | AL355304.1 |
| MIR1250 | CYP4F22 | MYO5C | SAA2 | AC092666.1 | MMP21 |
| AL031258.1 | HLA-DQA2 | CDKN2D | IGKV1D-12 | AL845472.1 | CA3-AS1 |
| AP003068.4 | AC136475.3 | SEM1 | ASB11 | FAT2 | AL121929.2 |
| LINC01269 | GP6 | CACNA1F | CPNE6 | IGLV4-69 | SPON1 |
| MIR4513 | WASHC1 | ULBP3 | RNU1-124P | GLDN | ZNF549 |
| SLC6A14 | SPDYE2B | RSPH9 | AC008074.1 | SCN3A | KLHL33 |
| AL137140.1 | SLC2A14 | TRIM16 | CIDEC | BRINP1 | ZNF483 |
| CST5 | AC131934.1 | CCDC96 | IGKV2-24 | AC079601.2 | BCL11B |
| MGAT3-AS1 | AC069148.1 | WASH4P | LINC00484 | TRIM17 | RAB9B |
| TMPRSS11E | MGAT3 | TMED3 | AC109630.1 | AL021155.4 | TMEM150C |
| AL592211.2 | TGFBI | AC018904.1 | AC018797.2 | LRRC37A7P | CACNA2D1 |
| AL008635.1 | AL118505.1 | ALG1L2 | RN7SL862P | MSH4 | FILIP1 |
| AC022079.1 | H2AC8 | EZR | IGLV10-54 | RNU6-1016P | NFKBIZ |
| Y_RNA | CDH2 | GIPC1 | AC004009.2 | ADAMTS9-AS1 | THRB |
| NDUFB4P11 | CST2 | FTH1P7 | SLC26A4 | AL365361.1 | FAIM2 |
| MIR193A | CD55 | CCDC146 | AL773545.3 | DNM1P46 | AL731566.2 |
| AC093515.1 | NECTIN4 | TRIP10 | TRBJ1-3 | LINC00943 | C3orf70 |
| DRC1 | H2BC4 | MRPS6 | AL136366.1 | OTOA | CD302 |
| ACTG1P14 | PKD2L1 | Z92544.1 | AL160291.1 | COL19A1 | AC005034.5 |
| PTPRZ1 | MFSD2A | LINC02166 | UBE2CP2 | WDR45BP1 | FMO1 |
| KRT16 | SNORC | MVD | AL355852.1 | BX293535.1 | CD160 |
| AL110115.2 | AGMO | FTH1P8 | IGLV1-41 | LINC01410 | PDE5A |
| PHACTR3 | ANK1 | ENO1 | IGKV2D-24 | CHST2 | PYGM |
| LINC02100 | LINC00923 | CHPF | AC098679.5 | FCER1A | TNRC6C |
| HES5 | SLCO4A1-AS1 | CATSPERG | CASC19 | KCNA3 | MAP3K4-AS1 |
| CST6 | THORLNC | DUSP14 | UGT1A8 | CA3 | ZDBF2 |
| AC115099.1 | KRT18P15 | RCCD1 | CCDC168 | CAMK4 | RSPH4A |
| RN7SL541P | B3GALT5 | IFT22 | DNAH8 | AC097493.3 | ZNF682 |
| AC110769.3 | BX640514.2 | NSDHL | ATP5MF-PTCD1 | KLHL41 | NBEA |
| ELF5 | H1-4 | CLTB | DAZL | ITGA8 | MAP3K2-DT |
| LINC02321 | BX470102.1 | FOXD2-AS1 | IGF1 | RASGEF1A | ZFHX4 |
| GAS2L2 | SSC4D | PPP1R14BP3 | JCHAIN | TMSB15B-AS1 | COQ8A |
| AC018553.1 | LINC02014 | ENDOG | RNU6-1165P | AC091588.3 | KIF21B |
| CDA | SCGB3A2 | HMGB1P1 | SLC5A5 | NTRK2 | TRGV7 |
| AC131888.1 | AL078622.1 | RASAL1 | FABP4 | SLC8A3 | ZNF681 |
| LINC01748 | IL1RN | WDR54 | AL118523.1 | RASL11A | ESR1 |
| AF228730.5 | PTMAP9 | RTN4R | AC104779.1 | GUSBP17 | RGS9 |
| MROH7-TTC4 | IGFBP2 | PPIAP11 | AL133406.1 | LRRN3 | PPP1R12B |
| DNAH11 | CDRT15P1 | PFKFB4 | BHLHB9 | CYP3A4 | ZHX1 |
| MAB21L4 | UBQLNL | CIB1 | IGKJ4 | GTF2H2B | PCDHB7 |
| MIR6859-4 | PLAT | DTYMK | TCL1A | AC010463.3 | SETBP1 |
| KLK11 | AP000525.1 | IER5L | IGHJ4 | ANK2 | AL158163.1 |
| ATP6V0CP4 | DTX2 | SNRPA1P1 | LEP | IGSF10 | AGAP2 |
| LINC02470 | PSMA6P2 | CEP170B | LINC00671 | AL109614.1 | RNF150 |
| SLC26A9 | IFI30 | PFDN2 | ALG1L8P | FAM131B | CCDC188 |
| AL513318.1 | AC006213.7 | ACOT1 | DUOXA2 | AC012435.3 | CDC14C |
| AC133785.1 | LRAT | OSGIN1 | LINC01215 | COL25A1 | SMARCA5-AS1 |
| CFAP47 | NT5DC4 | FAH | TRBV23-1 | AC004908.3 | ZNF737 |
| LYPD3 | AC241377.4 | CTNNBIP1 | AL161630.1 | ELANE | RBPMS2 |
| LINC01929 | AC009054.2 | TMEM187 | SAA1 | PARP11-AS1 | FAM198B-AS1 |
| CSGALNACT2P1 | AL031668.2 | SH3BGRL3 | BNIP3P26 | LRRC55 | AC007842.1 |
| AC106045.1 | AC010207.1 | RAMACL | LRRC77P | FAM163A | AC008555.2 |
| AC135983.3 | NQO1 | VAMP8 | IGKV2-28 | AC010761.3 | FAM66B |
| GJB5 | SEMA3B | HECW2-AS1 | IGKV1-37 | ZNF724 | AC107959.1 |
| SLC4A11 | PKM | RHEBP2 | NSA2P3 | ADGRE4P | GUCY1A1 |
| AL355102.1 | AC068580.4 | PPP2R5B | AL671883.1 | AC008149.2 | LINC02019 |
| ADGRF2 | PRSS27 | NIBAN2 | AMPD1 | TAFA2 | ROR1 |
| ALDH3B2 | SCAT1 | ZNHIT2 | IGLV7-43 | DGAT2 | GPC6 |
| S100A4 | AC005551.1 | FTSJ1 | C12orf71 | GPR174 | TOX2 |
| AL451166.1 | ANXA2P1 | PDXP | AC243829.1 | RPS6KA6 | BTG2 |
| H3C1 | GRIN1 | ARPC3P1 | AC006059.5 | AC002525.1 | PDE7A |
| KISS1 | CRIP1 | MRPL2 | CHRDL2 | RNF217-AS1 | PRKN |
| CU639417.5 | AC108134.1 | RFC2 | CEACAM7 | RN7SL181P | GPAM |
| AL157373.2 | IMPDH1P10 | TUBB4B | AC245100.3 | GP5 | KCNN3 |
| C9orf24 | AC005753.1 | ARPC1A | NGF-AS1 | BHLHE22 | AP000648.4 |
| SLURP1 | COL22A1 | PPDPF | IGHV2-70 | EDNRB | MPP6 |
| AC006237.1 | AL512274.1 | LRRC23 | AC116345.1 | AC090948.3 | SLC2A13 |
| SIX3-AS1 | SCT | VPS9D1 | AC025470.2 | NEGR1 | ZNF596 |
| AC002306.1 | P3H2 | AP4M1 | INTS9-AS1 | LRRC2 | RGS5 |
| KRT17P2 | DISP2 | VIM | AC012213.1 | FHL5 | CGREF1 |
| DLG1-AS1 | VEGFC | FTH1P11 | ANAPC1P1 | CNR1 | AC096992.2 |
| PRODH | MROH6 | COL13A1 | WNT1 | AC016588.2 | AC102945.2 |
| FP671120.2 | NOL3 | STC2 | LINC01797 | AC005332.2 | TTLL7 |
| TFAP2A-AS1 | PGGHG | BCL2L1 | LINC00939 | AC083843.3 | LRRC8C-DT |
| METTL11B | AL034417.4 | PDIA3 | AC006452.1 | TMEM30A-DT | SLC4A7 |
| LINC01956 | SMCO2 | NOP10 | LINC01882 | AL512625.3 | HCG11 |
| CLIC3 | ADAMTSL5 | PSME2P2 | C10orf105 | LYVE1 | ZBTB10 |
| SFTA1P | SUGCT | ZFYVE19 | LINC02847 | ANKRD29 | MAN1A1 |
| SFTA2 | CCDC9B | BX255925.3 | CBLN4 | AP001636.3 | ABHD3 |
| CFAP77 | SPTBN2 | GDPGP1 | CLEC18C | MIR3142HG | FAM126A |
| SERPINB2 | RHBDL1 | ATG4B | DBX2 | AC092645.1 | ADAMTS9 |
| PSORS1C2 | H3C6 | PHPT1 | RNA5SP111 | HSD17B13 | SNHG14 |
| P3H2-AS1 | ANXA2P2 | ATF5 | C6orf58 | ANGPT1 | CRISPLD2 |
| RHOV | KYNU | ATP5MF | CLC | AC090617.5 | REV3L |
| AL354953.1 | BICDL2 | BCAR1 | TLX1 | AL590064.1 | GLCCI1 |
| PKD1P2 | ADAP1 | EFNA4 | AL353704.1 | ASAH2 | ZNF43 |
| FAM183A | EPHA4 | RMDN3 | LINC02397 | AL161457.2 | PAXBP1-AS1 |
| TBX15 | BATF2 | SAMD10 | TRBV12-3 | AC119396.1 | LRRC8C |
| LEMD1 | ANXA2 | ERG28 | IGLV3-16 | DCLK3 | SULT1C4 |
| HCG4B | PDE6A | COQ2 | IGLV1-51 | SNRK-AS1 | SCARA3 |
| ACOT6 | AC006077.2 | ARRDC1 | OPA1-AS1 | CNNM3-DT | ITGA9 |
| CCDC114 | NFE2 | EDF1 | TRAV8-1 | KCNG2 | RHOBTB1 |
| AC005392.2 | PCDHB13 | HPCAL1 | AL583785.1 | ARL9 | LINC00680 |
| SYT8 | AC106028.3 | TIMM17B | AL731769.2 | LAX1 | AC067750.1 |
| NCCRP1 | EFNA3 | FAM207BP | AC009812.3 | KCND3 | B4GALT6 |
| KCNMB2-AS1 | TMEM191A | BUD31 | LINC01973 | CXCL1 | ADGRL2 |
| FGF16 | CRNDE | AAGAB | LINC02461 | WNT10B | ZIK1 |
| FOXCUT | CDC25C | ZNF487 | VWDE | NNAT | GCSAM |
| ZNF341-AS1 | CAP1P1 | BUD23 | PART1 | KCNA2 | AVIL |
| AC008758.4 | AC092171.1 | BCL7C | IFITM3P1 | AC113189.3 | SLC9A9 |
| MT3 | CLDN4 | VKORC1 | AC060834.1 | AC025034.1 | TNKS |
| CTNNA1P1 | RPL17P50 | MED11 | KCNIP2-AS1 | AC022509.2 | GPR75 |
| AC025062.1 |  |  | AP005131.5 | KIF6 | KAT2B |
|  |  |  | LEFTY2 | ZNF208 | ZNF578 |
|  |  |  | TRBV14 | AL355916.2 | PTPRM |
|  |  |  | BMP3 | AC004908.1 | SHPRH |
|  |  |  | FCRL1 | PRSS35 | OSBPL1A |
|  |  |  | GAL | DOCK11P1 | AC007191.1 |
|  |  |  | AC139493.2 | IL33 | EPAS1 |
|  |  |  | AL133467.1 | LRFN5 | PIK3R1 |
|  |  |  | AC004846.2 | CLGN | AC016596.2 |
|  |  |  | AC016910.1 | AL844892.2 | C7orf31 |
|  |  |  | LINC01894 | AC009242.1 | ARHGAP6 |
|  |  |  | TEX26-AS1 | ADD3-AS1 | KCND1 |
|  |  |  | PRDM12 | AL137009.1 | AP000766.1 |
|  |  |  | IGLV2-18 | GIMAP5 | ZNF658 |
|  |  |  | AC140479.3 | CYP26B1 | CEP85L |
|  |  |  | LINC00861 | WDR17 | TACC1 |
|  |  |  | IGLV4-3 | MIR186 | CUBN |
|  |  |  | XPNPEP2 | LINC00624 | TBC1D4 |
|  |  |  | TRAV27 | TSPEAR-AS1 | ITSN1 |
|  |  |  | SCN2A | FAM13C | AC021087.2 |
|  |  |  | IGHM | SAMD5 | ZNF852 |
|  |  |  | PFN1P8 | AC233992.3 | SASH1 |
|  |  |  | IGKV3D-20 | LPAR4 | RPL23AP53 |
|  |  |  | MIR1-1HG-AS1 | ITK | AC018413.1 |
|  |  |  | TRBV28 | RASD1 | SYNE3 |
|  |  |  | NT5C3AP2 | CRISPLD1 | TRIL |
|  |  |  | LINC01819 | NMUR1 | IL6ST |
|  |  |  | PLIN4 | AC017071.1 | ABCC4 |
|  |  |  | CTD-2297D10.2 | AC006059.1 | TTC28 |
|  |  |  | AL360181.1 | MAPK4 | CDC14A |
|  |  |  | E2F3P1 | SNAP25 | NIPSNAP3B |
|  |  |  | PPP4R4 | SELENOP | HAUS6 |
|  |  |  | EYA1 | AL139288.1 | RPL32P3 |
|  |  |  | SFRP1 | AL359878.1 | FOXO3B |
|  |  |  | PLSCR2 | SMG1P4 | TNFSF14 |
|  |  |  | GABRA4 | AL162171.1 | NKTR |
|  |  |  | AL513190.1 | CILP | CPEB3 |
|  |  |  | TRBJ1-4 | EPHX3 | MFHAS1 |
|  |  |  | CYCSP24 | EBF2 | RAVER2 |
|  |  |  | LINC02688 | PGM5P2 | AC012063.1 |
|  |  |  | TRBJ1-5 | IKZF3 | ERI1 |
|  |  |  | CBLN1 | RGL4 | FSD1L |
|  |  |  | AL096816.1 | AC006504.7 | STOM |
|  |  |  | SILC1 | PLEKHH2 | ZNF605 |
|  |  |  | AL451123.2 | NCR3LG1 | AC093827.4 |
