# Supplementary material for: Heterogeneity of weight loss and transcriptomic signatures in pancreatic ductal adenocarcinoma
Source: J Cachexia Sarcopenia Muscle. 2023 Dec 20;15(1):149–58. doi: 10.1002/jcsm.13390 (PMC10834348; doi:10.1002/jcsm.13390)
Supplement: Supplementary file 4 — Table S4. Lists of differentially expressed genes in cachectic PDAC patients with pancreatic head tumours compared to cachectic PDAC patients with distal pancreatic tumours (p < 0.05, fc < −1.5 or fc > 1.5). [file JCSM-15-149-s002.docx]

| **Ingenuity Canonical Pathways** | **-log(p-value)** | **z-score** |
| --- | --- | --- |
| Role of Hypercytokinemia/hyperchemokinemia in the Pathogenesis of Influenza | 2.35 | -2.83 |
| LPS/IL-1 Mediated Inhibition of RXR Function | 3.53 | -2.12 |
| Pathogen Induced Cytokine Storm Signaling Pathway | 4.58 | -2.12 |
| TREM1 Signaling | 4.72 | -2.11 |
| IL-8 Signaling | 2.73 | -1.94 |
| Hepatic Fibrosis Signaling Pathway | 6.12 | -1.73 |
| Role Of Chondrocytes In Rheumatoid Arthritis Signaling Pathway | 4.01 | -1.60 |
| Differential Regulation of Cytokine Production in Macrophages and T Helper Cells by IL-17A and IL-17F | 3.82 | -1.34 |
| Tumor Microenvironment Pathway | 3.44 | -1.29 |
| Osteoarthritis Pathway | 6.05 | -1.21 |
| Cardiac Hypertrophy Signaling (Enhanced) | 3.91 | -1.18 |
| Type II Diabetes Mellitus Signaling | 2.16 | -1.13 |
| S100 Family Signaling Pathway | 6.41 | -1.13 |
| Neutrophil Extracellular Trap Signaling Pathway | 2.60 | -1.04 |
| Role Of Osteoclasts In Rheumatoid Arthritis Signaling Pathway | 3.71 | -0.85 |
| Factors Promoting Cardiogenesis in Vertebrates | 3.11 | -0.83 |
| Differential Regulation of Cytokine Production in Intestinal Epithelial Cells by IL-17A and IL-17F | 4.32 | -0.82 |
| Pulmonary Healing Signaling Pathway | 2.96 | -0.78 |
| Role Of Osteoblasts In Rheumatoid Arthritis Signaling Pathway | 2.90 | -0.73 |
| Role of MAPK Signaling in Inhibiting the Pathogenesis of Influenza | 2.58 | -0.71 |
| Wound Healing Signaling Pathway | 4.05 | -0.45 |
| Pulmonary Fibrosis Idiopathic Signaling Pathway | 3.00 | -0.45 |
| Phospholipases | 2.32 | -0.38 |
| GPCR-Mediated Nutrient Sensing in Enteroendocrine Cells | 2.02 | -0.33 |
| RAR Activation | 2.85 | -0.20 |
| Phagosome Formation | 2.63 | -0.17 |
| ILK Signaling | 2.08 | 0.30 |
| Serotonin Receptor Signaling | 3.98 | 0.37 |
| Inhibition of Matrix Metalloproteases | 2.21 | 0.45 |
| Gαq Signaling | 2.69 | 0.91 |
| Antioxidant Action of Vitamin C | 2.62 | 1.00 |
| Oxytocin In Brain Signaling Pathway | 2.52 | 1.07 |
| Human Embryonic Stem Cell Pluripotency | 2.48 | 1.07 |
|  |  |  |
| **Supplemental Table 3.** **Enriched Pathways in PDAC Tumor Transcriptomes Among Cachectic Patients by Tumor Anatomic Location.** Ingenuity Pathway Analysis (Qiagen®) data are presented for cachectic patients with pancreatic head tumors compared to cachectic patients with distal pancreatic tumors. Pathways significantly inactivated (negative z-score) and activated (positive z-score) in pancreatic head tumors from cachectic patients are reported (p-value ≤ 0.01 or -log(p-value) ≥ 2.0). | | |
